# Supplementary material for: The ultrafast onset of exciton formation in 2D semiconductors
Source: Nat Commun. 2020 Oct 19;11:5277. doi: 10.1038/s41467-020-18835-5 (PMC7572483; doi:10.1038/s41467-020-18835-5)
Supplement: Supplementary file 2 — Supplementary Information [file 41467_2020_18835_MOESM2_ESM.pdf]

Supplementary Information

The ultrafast onset of exciton formation in 2D semiconductors

Trovatello et al.

Supplementary Note 1: Static characterization

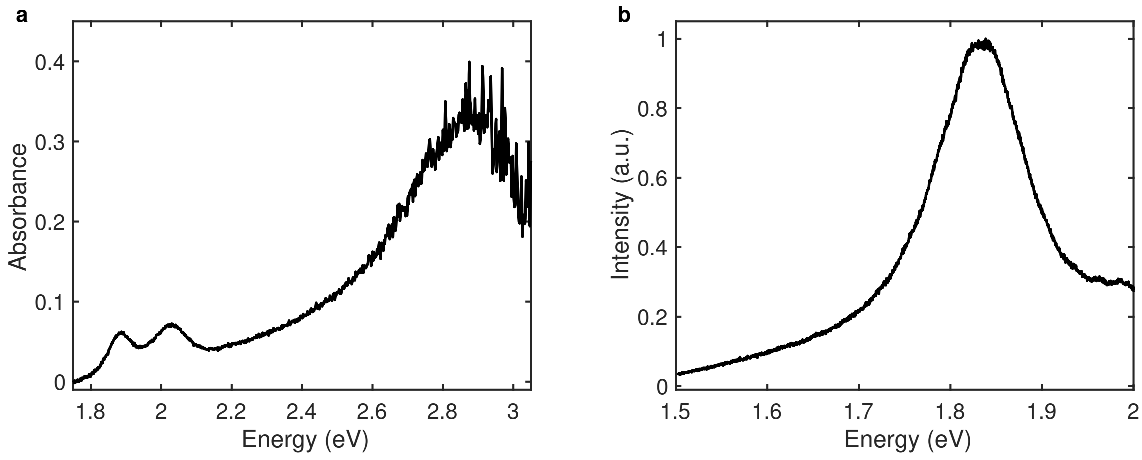

Supplementary Figure 1: **Static characterization.** a) Static absorbance and b) PL spectrum of 1L-MoS_2, T=300 K.

The absorption spectrum in Supplementary Fig. 1a, is obtained from a reflective contrast measurements. The reflectivity from the sample (R_{MoS2}) and the substrate (R_{sub}) are separately collected and the reflectivity contrast R_{contr} is calculated as (R_{MoS_2}-R_{sub})/R_{sub}. Kramers-Kroening analysis [1] is used to fit the reflectivity contrast and to extract the absorption spectrum A=0.25 $R_{contr}(n_s\text{-}1)$, where n_s is the refractive index of the substrate. The photoluminescence spectrum is measured at T=300 K with 2.5 eV excitation. The spectrum is dominated by the A exciton emission at ∼1.86eV (Supplementary Fig. 1b).

Supplementary Note 2: Exciton population dynamics

As reported in the main text, the non-equilibrium optical response of TMDs is the result of the transient modification of the excitonic peaks induced by photo-excitation processes. The physical processes can be categorized as many-body and population effects. In the former process, photoexcited carriers transiently enhance the Coulomb screening leading to a renormalization of the exciton binding energy and transient shift of the excitonic resonances. In the latter process, photoexcited carriers induce a transient reduction of the exciton oscillator strength due to phase space filling. These effects are expected to happen on an extremely fast (i.e. sub-100 fs) timescale and can be, in principle, be disentangled

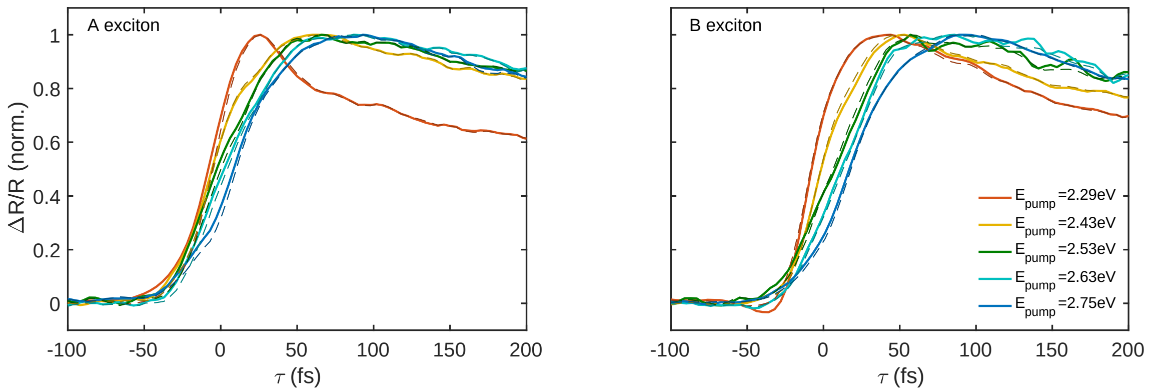

Supplementary Figure 2: **Pauli Blocking.** ΔR/R dynamics taken at the maximum of the bleaching signal (continuous lines) and by spectral integration (dashed lines) around the A exciton (left panel) over a bandwidth of 57 meV (20 nm), and the B exciton (right panel) over a bandwidth of 70 meV (20 nm). All the traces are normalized.

by a careful line shape analysis of the exciton peak dynamics. An alternative way to single out population effect consists in integrating the ΔR/R map on a broad energy range around the excitonic resonances [2]. The pump-probe signal obtained by such a spectral integration does not depend on any energy shift and it is proportional to the exciton population density. In Supplementary Fig. 2 we compare the ΔR/R dynamics measured at the maximum of the bleaching signal and the dynamics of the traces obtained by spectral integration around the exciton bleaching signal. For all the excitation energies, after renormalization, the traces almost overlap. This is a clear indication that the ΔR/R bleaching signals on resonance with the excitonic peaks are mainly determined by the population effect, while many-body effects play a minor role.

Supplementary Note 3: Exciton dynamics upon UV pump excitation

In Supplementary Figure 3, we reported the ΔR/R formation dynamics measured upon excitation with a pump pulse centered around 3.75 eV, i.e. almost 1 eV higher than the maximum value of the pump energy reported in the manuscript. In this experiment, the UV pump pulses are obtained by frequency up-conversion of a broadband NOPA as explained in details in Ref. [3]. These pulses are compressed down to 20 fs by a pair of prisms and temporally characterized by a two-dimensional spectral-shearing interferometry method

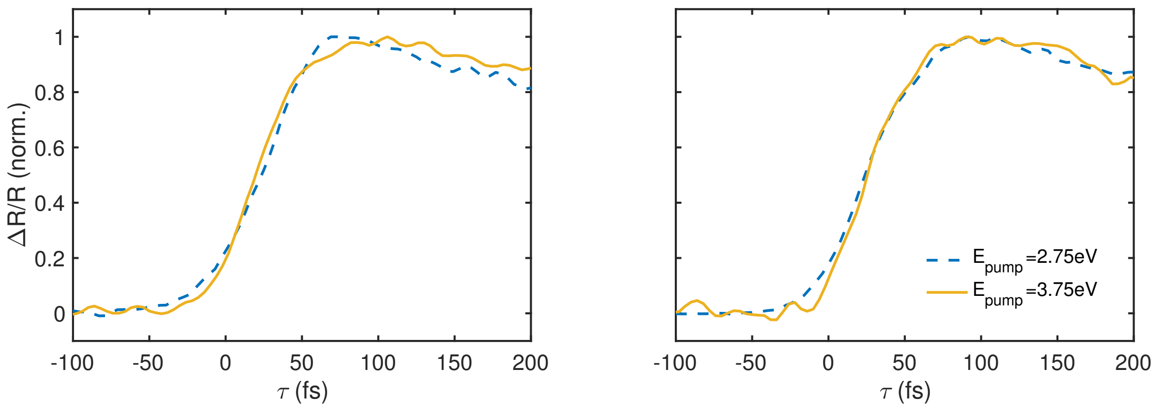

Supplementary Figure 3: **UV photon energy excitation.** ΔR/R traces measured across A_{1s} (left panel) and B_{1s} (right panel) excitonic resonances for E_{pump}=3.75 eV (yellow lines). In both graphs we report the ΔR/R traces at E_{pump}=2.75 eV for comparison (dashed lines). For both the excitation conditions the two dynamics have almost the same build-up time.

(2DSI) in the UV region. As shown in the figure, τ_{rise} do not follow the same trend observed up to 2.75 eV and it seems to maintain a constant value between 30-40 fs. We fit the rising edge dynamics with the same fitting function described in the main text and we obtain the following values for τ_{rise} of A_{1s} and B_{1s} excitons: 34 ± 3 fs and 37 ± 5 fs, respectively. This result points towards a possible saturation effect of the exciton formation dynamics at higher energies. Unfortunately, we are not able to perform pump-probe experiments with sub-30 fs temporal resolution in the broad energy window ranging from 2.75 eV and 3.75 eV and to give a precise estimation of the energy where this saturation effect sets in. When the laser pulse is tuned to such a high energy, optical transition involving electronic states close to Γ and M symmetry points can be activated in addition to the optical transitions around the K/K' points. We can tentatively explain the deviation of the exciton formation time from the monotonic increase trend observed at lower pump energies, as a result of a Pauli blocking effect due to the quick scattering of photoexcited carriers from different symmetry points (i.e. Γ and M) of the Brillouin zone to the K/K' points. These scattering processes are mediated by large-momentum acoustic phonons. Moreover, 3.75 eV is almost two times the exciton energy. At this excitation energy, also Auger-like relaxation processes (i.e. 1 high energy exciton is annihilated while 2 A/B excitons are created) could come into play, possibly leading to a faster relaxation of the excitons towards the band edge.

Supplementary Note 4: Transient optical response for long delay time

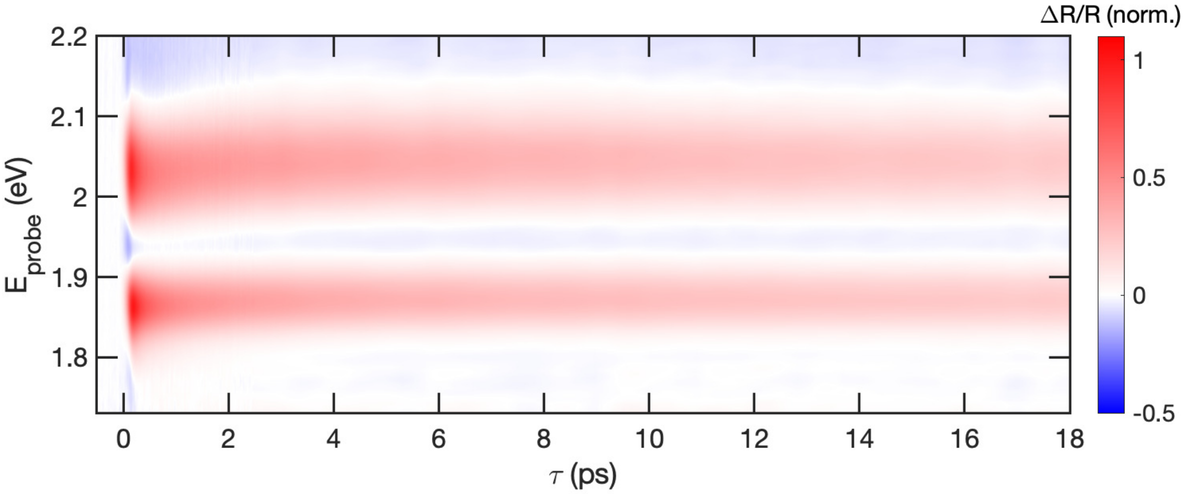

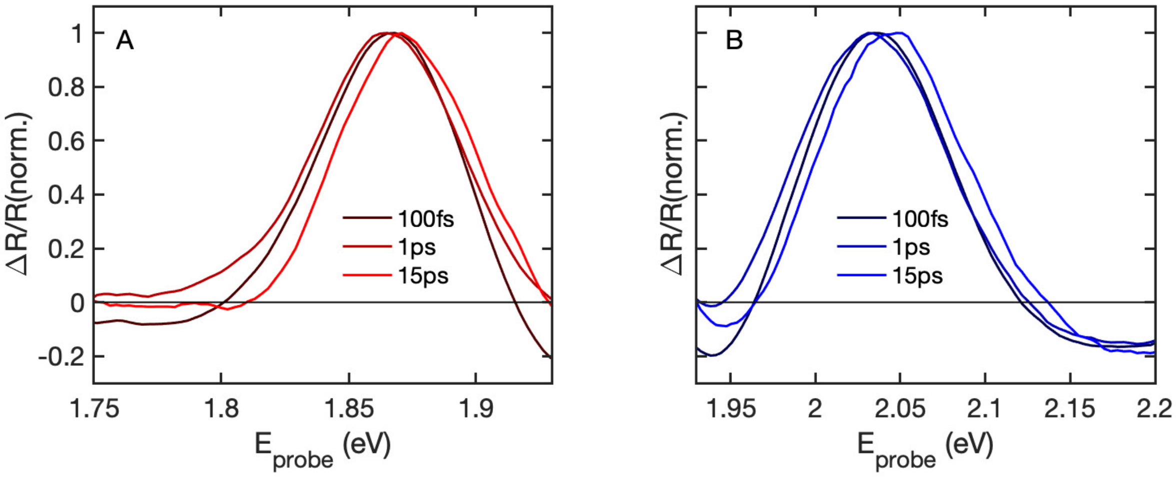

Supplementary Figure 4: **Long dynamics.** ΔR/R map measured on 1L-MoS_2 at long delay time following an excitation photon energy of 2.43 eV. Close-up of the ΔR/R normalized spectra around the A_{1s} (bottom left) and B_{1s} (bottom right) excitonic transition at τ =100 fs, 1ps, 15 ps. The two normalized traces at 100 fs and 1 ps have the same shape and are centered at the same energy while the trace at longer delay is blue-shifted by ~10 meV. A transient energy shift of the ΔR/R spectrum observed at longer delay time is a signature of additional effects such as thermal heating.

Different physical processes can lead to a transient modification of the excitonic resonances and determine the non-equilibrium optical response at early delay times. Coulomb

many-body effects such as bandgap renormalization or biexcitons [4] renormalize the exciton energies leading to a shift of the exciton resonances. Differently, Pauli blocking causes the reduction of the exciton oscillator strength due to phase-space filling. These effects are difficult to disentangle and their combination might result in a varying profile of the ΔR/R spectrum for different time delays [5]. At longer delays, photoexcited excitons/carriers release their excess energy to the lattice. The consequent increase of the lattice temperature results in an energy shift of the excitonic resonances [6]. This effect manifests as a few meV energy shift of the ΔR/R peak occurring on a ps timescale (see Supplementary Fig. 4) and therefore can be disregarded in the analysis of the early delay transient signal.

Supplementary Note 5: Pulse characterization

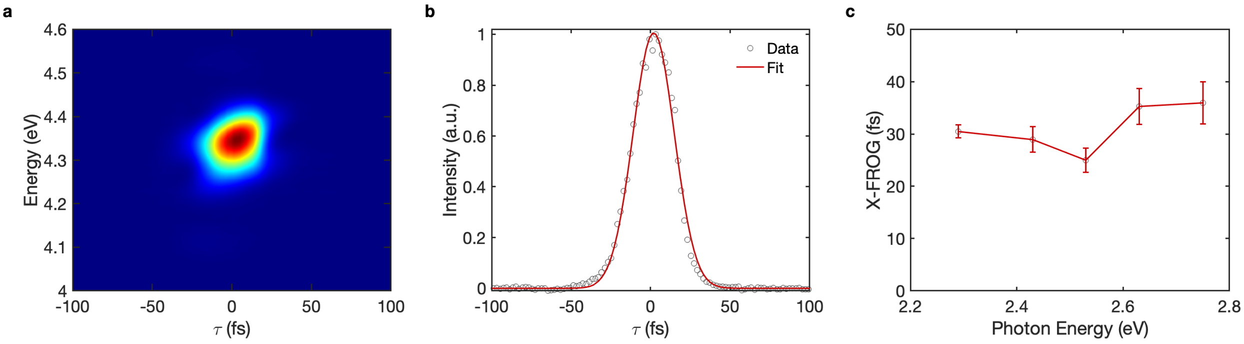

Supplementary Figure 5: **X-FROG.** a) X-FROG map of the pump and probe pulses used in the transient reflectivity experiments. The map is generated by acquiring the sum frequency spectrum of the pump and probe pulses, at different delay times. b) (Black dots) Delay marginal of the X-FROG map. (Red curve) Fit of the temporal profile to a single Gaussian function. The FWHM of the fitting function is the estimation of the temporal resolution. c) FWHM of different X-FROG traces obtained by tuning the energy of the pump. The error bars represent the standard deviation of the width values obtained performing several pulse characterizations.

As extensively reported in the main text, the build-up dynamics of the ΔR/R traces can provide a direct estimation of the formation time of the incoherent excitons. In order to get insight into the intrinsic timescale of this process, it is crucial to carefully estimate the temporal resolution of the setup. This is accomplished by performing Cross-Frequency Resolved Optical Gating (X-FROG) measurements. The pump and the probe beams are

focused on a very thin (i.e. 10 μm) BBO crystal which is placed at the same position of the MoS_2 sample. The sum-frequency spectrum generated in the non-linear crystal is detected by CCD camera at different delay times between the pump and the probe. The resulting X-FROG map is reported in Supplementary Fig. 5a. The full width at half maximum (FWHM) of the cross-correlation trace obtained by integrating over the energy of the sum-frequency spectrum (Supplementary Fig. 5b) represents an upper bound of the temporal resolution of the experiment. This characterization is performed for each pump pulse (Supplementary Fig. 5c) before and after measuring the transient reflectivity map. The time t=0 of the pump-probe experiments is defined by the maximum of the X-FROG traces.

Supplementary Note 6: Fitting procedure

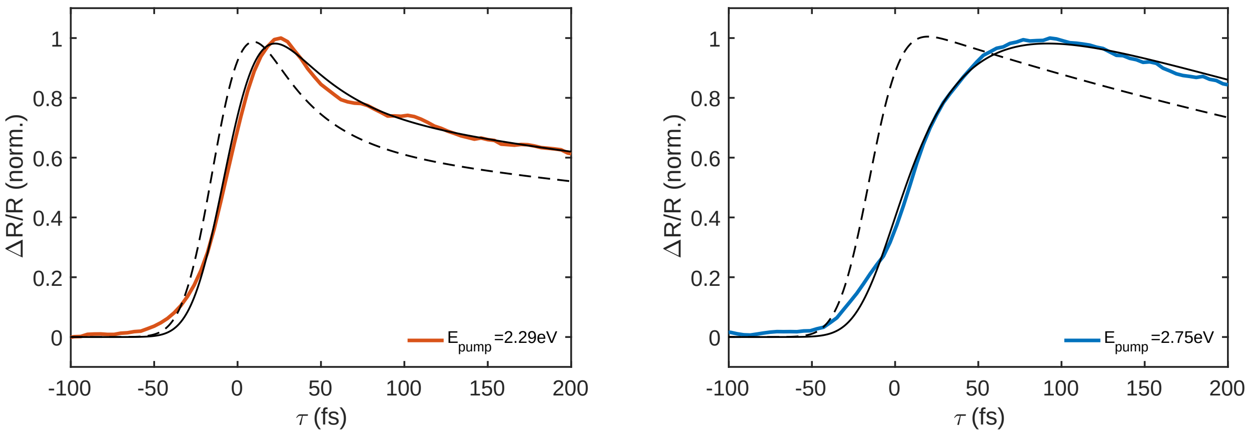

Supplementary Figure 6: **Fitting procedure.** The colored lines are the temporal cuts of the ΔR/R map measured at the A exciton resonance for low (left panel) and high (right panel) pump photon energy excitation. The black continuous lines are the fit to the data. The black dashed lines are the fitting curves with τ_{rise}=0.

As reported in the main text, the ΔR/R time traces are fitted by a rising exponential, describing the build-up of the transient signal, and two exponential decays related to the different timescales of the exciton relaxation processes. This fitting function is convoluted with a Gaussian function accounting for the finite instrumental response function (IRF). The results of the fit are reported in Supplementary Fig. 6 for the traces measured with the lowest and highest pump photon energies. The IRF is carefully determined by cross-correlation

frequency resolved optical gating (X-FROG) measurements (see previous section). The dashed lines in Supplementary Fig. 6 are the fitting functions without the contribution of the rising exponential, i.e. assuming an instantaneous response. It is clear that only by including the effect of a finite exciton formation time in the fitting function, the ΔR/R traces at the early femtosecond timescale can be satisfactorily reproduced. The ΔR/R trace at lower pump energy displays an almost instantaneous rise time (τ_{rise}=12±4 fs) and a bi-exponential decay behavior. According to the simulations, the fast time constant τ_1=32±6 fs represents the timescale for the exciton polarization to population transfer, while τ_2 is attributed to decay process of the exciton population and it is on the order of few ps. The ΔR/R trace at higher pump energy displays a single ps decay component and a longer rise time (τ_{rise}=29±3 fs). For this excitation condition, the ΔR/R signal is dominated by the incoherent exciton population. In this case, the finite build up time is a solid and direct estimation of the incoherent exciton formation process.

Supplementary Note 7: Polarization and fluence dependence

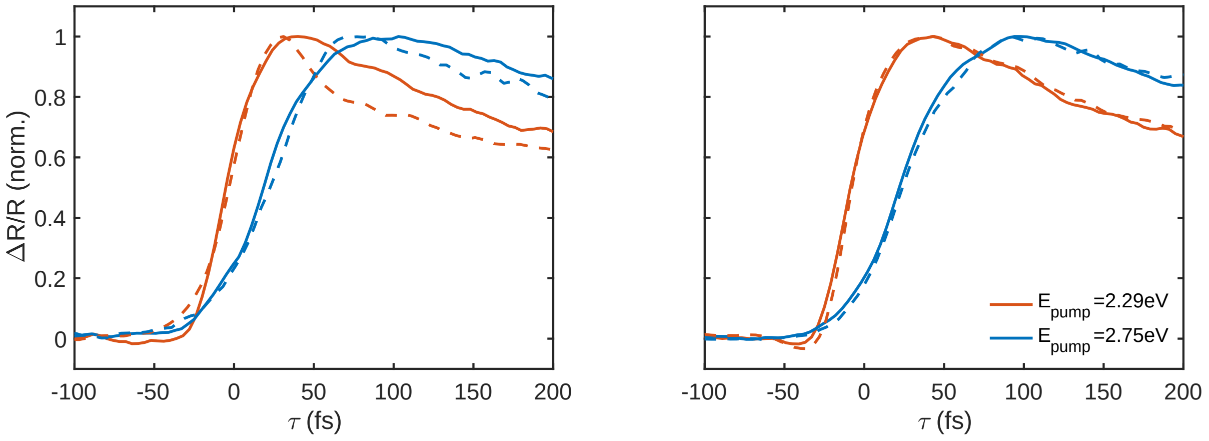

Supplementary Figure 7: **Polarization dependence.** Normalized ΔR/R traces measured at A_{1s} (left) B_{1s} (right) at different pump polarizations, and pump photon energy excitations, i.e., 2.29 eV (orange) and 2.75 eV (blue). The probe polarization is set to be linear-vertical, while the pump is linear-horizontal (continuous lines), and linear-vertical (dashed lines).

The build up dynamics of A_{1s} and B_{1s} exciton transitions does not display any dependence on the pump and probe polarizations as reported in Supplementary Fig. 7 and on

the excitation fluence as reported in Supplementary Fig. 8).

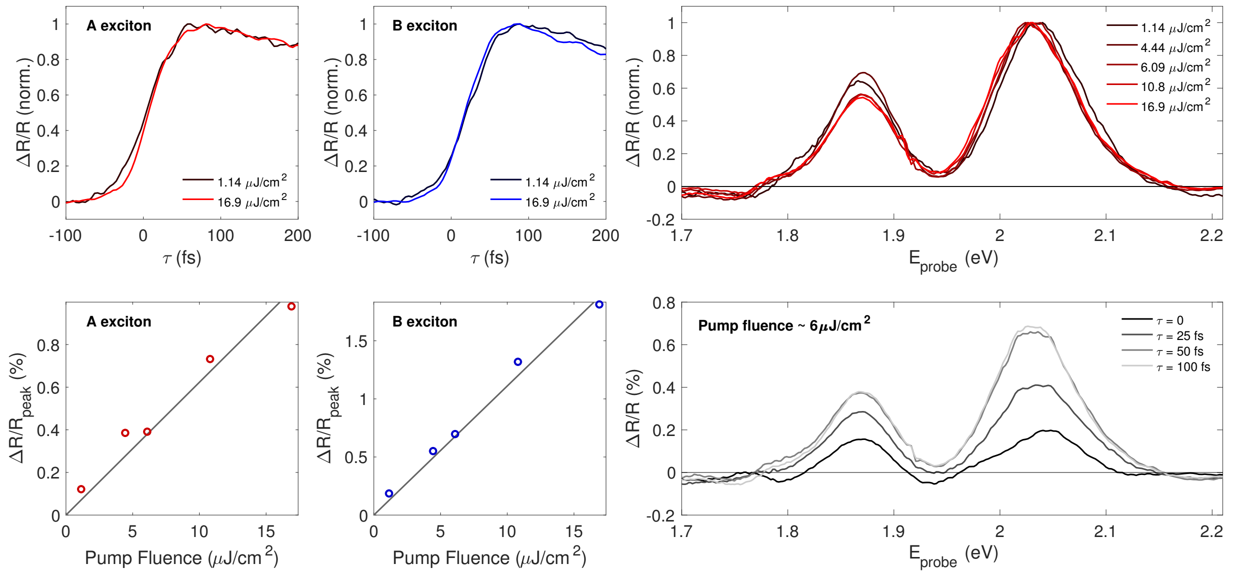

Supplementary Figure 8: **Pump fluence dependence.** Normalized ΔR/R dynamics (top left) and spectra (top right) measured at A_{1s}/B_{1s} excitonic resonances at different pump fluences in the range 1-20μJ/cm^2 with pump photon energy of 2.75 eV. The peak of A and B exciton bleaching signal displays a linear dependance with the pump fluence in the examined range (bottom left). The transient spectra at different time delays (bottom right) show the spectral profile of A and B excitons in the temporal region of explored in the experiment.

Supplementary Note 8: Excitons in 1L-TMDs

The equilibrium optical response of the TMD is characterized by the microscopic interband polarization $\langle c^{\dagger}_{\xi,s,q} v_{\xi,s,q}\rangle$, where $c^{\dagger}_{\xi,s,q}$ ($v_{\xi,s,q}$) generates a conduction (valence) band electron (hole) near the corner ξ = K, K$'$ of the first Brillouin zone with spin $s = \uparrow, \downarrow$ and two-dimensional in-plane wave vector q [7]. Solving the Wannier equation [8] accesses Coulomb correlated electron-hole excitations near the TMD band gaps:

$$\frac{\hbar^2 q^2}{2\mu_\lambda}\varphi_{\lambda,q} - \sum_{q'} \frac{V_{q'}}{\epsilon_{q'}}\varphi_{\lambda,q+q'} = (\epsilon_\lambda - \epsilon_g)\varphi_{\lambda,q}. \quad (1)$$

Here, $\mu_\lambda = m^e_\lambda m^h_\lambda/(m^e_\lambda + m^h_\lambda)$ denotes the reduced mass defined with respect to the effective masses of electrons (holes) in the conduction (valence) band m^e_λ (m^h_λ) [9]. $V_{q'}/\varepsilon_{q'}$ describes the screened Coulomb potential which includes the bare Coulomb potential $V_{q'}$ accounting for finite thickness effects [10] and the non-local dielectric function $\varepsilon_{q'}$ parameterized from density function theory calculations [11]. The analytical model $\varepsilon_{q'}$ of the dielectric function characterizes the dielectric environment with a non-linear q-dependence. Due to the fact that excitons in TMDCs are stable over a wide range of excitation power and doping level [12, 13], treating the Coulomb potential statically captures the investigated excitonic effects. The evaluation of the Wannier equation (1) provides a set of bound and continuum solutions $\varphi_{\lambda,q}$ with energies ϵ_λ [14, 15]. The compound index λ comprises the high symmetry point ξ = K, K$'$ and spin $s = \uparrow, \downarrow$ of the involved electron and hole as well as the exciton state (bound and continuum states). This enables to work in a convenient excitonic basis including coherent exciton transitions P_λ, biexcitons and exciton-exciton scattering states B_η, as well as incoherent exciton populations $N_{\lambda_1,\lambda_2,q}$. The exciton dephasing is chosen equivalent to the energetically lowest A_{1s}/B_{1s} state which has been adjusted to the experimentally measured absorption spectrum: $\gamma_{A_\nu} = \gamma_{A_{1s}} = 39$ meV/$\hbar$, $\gamma_{B_\nu} = \gamma_{B_{1s}} = 66$ meV/$\hbar$.

SUPPLEMENTARY REFERENCES

[1] Kuzmenko, A. B. Kramers-Kronig constrained variational analysis of optical spectra. *Rev. Sci. Instr.* **76**, 083108 (2005).

[2] Berghäuser, G. et al. Inverted valley polarization in optically excited transition metal dichalcogenides. *Nat. Commun.* **9**, 971 (2018).

[3] Borrego-Varillas, R., Ganzer, L., Cerullo, G. & Manzoni, C. Ultraviolet Transient Absorption Spectrometer with Sub-20-fs Time Resolution. *Applied Sciences* **8**, 989 (2018).

[4] Sie, E. J., Frenzel, A. J., Lee, Y.-H., Kong, J. & Gedik, N. Intervalley biexcitons and many-body effects in monolayer MoS_2. *Phys. Rev. B* **92**, 125417 (2015).

[5] Aivazian, G. et al. Many-body effects in nonlinear optical responses of 2D layered semiconductors. *Npj 2D Materials and Applications* **4**, 025024 (2017).

[6] Ruppert, C. et al. The Role of Electronic and Phononic Excitation in the Optical Response of Monolayer WS_2 after Ultrafast Excitation. *Nano Lett.* **17**, 644–651 (2017).

[7] Katsch, F. et al. Theory of ExcitonExciton Interactions in Monolayer Transition Metal Dichalcogenides. *Phys. Stat. Solidi (b)* **255**, 1800185 (2018).

[8] Kira, M. and Koch, S. W. Many-body correlations and excitonic effects in semiconductor spectroscopy. *Progress in quantum electronics* **30**, 155–296 (2006).

[9] Kormanyos, A. et al. k·p theory for two-dimensional transition metal dichalcogenide semiconductors. *Npj 2D Materials and Applications* **2** 2, 022001 (2015).

[10] Latini, S., Olsen, T. & Thygesen, K. S. Excitons in van der Waals heterostructures: The important role of dielectric screening. *Phys. Rev. B* **92** (24), 245123 (2015).

[11] Trolle, M. L., Pedersen, T. G. & Véniard, V. Model dielectric function for 2D semiconductors including substrate screening. *Sci. Rep.* **7**, 39844 (2017).

[12] Steinhoff, A., Wehling, T. O. & Rösner, M. Frequency-dependent substrate screening of excitons in atomically thin transition metal dichalcogenide semiconductors. *Phys. Rev. B* **98**, 045304 (2018).

[13] Scharf, B., Van Tuan, D., Zutić, I. & Dery, H. Dynamical screening in monolayer transition-metal dichalcogenides and its manifestations in the exciton spectrum. *Journal of Physics:*

Condensed Matter **31**, 203001 (2019).

[14] Berkelbach, T. C., Hybertsen, M. S. & Reichman, D. R. Theory of neutral and charged excitons in monolayer transition metal dichalcogenides. *Phys. Rev. B* **88**, 045318 (2013).

[15] Qiu, D. Y., da Jornada F. H. & Louie, S. G. Optical Spectrum of MoS_2: Many-Body Effects and Diversity of Exciton States. *Phys. Rev. Lett.* **111**, 216805 (2013).
